# Pulmonary emphysema induced by methylphenidate: experimental study: Enfisema pulmonar induzido por metilfenidato: estudo experimental

**DOI:** 10.1590/1516-3180.2014.8470910

**Published:** 2014-11-28

**Authors:** Gabriel Victor Guimarães Rapello, Andréia Antoniolli, Daniel Martins Pereira, Gilberto Facco, Paulo Manuel Pêgo-Fernandes, Rogério Pazetti

**Affiliations:** I MSc. Physiotherapist, Hospital Regional de Mato Grosso do Sul, Campo Grande, Mato Grosso do Sul, Brazil.; II PhD. Associate Professor, Faculdade de Medicina (FAMED), Universidade Federal de Mato Grosso do Sul (UFMS), Campo Grande, Mato Grosso do Sul, Brazil.; III MSc. Physiotherapist, Hospital Regional de Mato Grosso do Sul, Campo Grande, Mato Grosso do Sul, Brazil, College Professor, Universidade Anhanguera-Uniderp, Campo Grande, Mato Grosso do Sul, Brazil.; IV MSc. College Professor, Universidade Anhanguera-Uniderp, Campo Grande, Mato Grosso do Sul, Brazil.; V MD, PhD. Full Professor, Instituto do Coração (InCor), Faculdade de Medicina da Universidade de São Paulo, São Paulo, Brazil.; VI PhD. Scientific Researcher, Laboratory of Thoracic Surgery Research, Faculdade de Medicina da Universidade de São Paulo (FMUSP), São Paulo, Brazil.

**Keywords:** Methylphenidate, Attention deficit disorder with hyperactivity, Pulmonary emphysema, Rats, Lung

## Abstract

**CONTEXT AND OBJECTIVE:**

Methylphenidate is the most widely used drug for treating attention deficit hyperactivity disorder. However, it has important side effects, such as abdominal pain, insomnia, anorexia and loss of appetite, and also some cases of early severe emphysema after drug abuse have been reported. Our aim was to investigate the development of pulmonary emphysema in rats that were subjected to different doses of methylphenidate.

**DESIGN AND SETTING:**

Experimental study carried out at the laboratory of a public university.

**METHODS:**

Eighteen male Wistar rats were divided into three groups: control (0.9% saline solution); MP 0.8 (methylphenidate, 0.8 mg/kg); MP 1.2 (methylphenidate, 1.2 mg/kg). After 90 days of daily gavage, the animals were sacrificed and lung tissue samples were prepared for analysis on the mean alveolar diameter (Lm).

**RESULTS:**

The Lm was greater in MP 0.8 (47.91 ± 3.13; P < 0.01) and MP 1.2 (46.36 ± 4.39; P < 0.05) than in the control group (40.00 ± 3.48).

**CONCLUSION:**

Methylphenidate caused an increase in the alveolar diameter of rats, which was compatible with human pulmonary emphysema.

## RESUMO

**CONTEXTO E OBJETIVO:**

O metilfenidato é o medicamento mais utilizado para o tratamento de déficit de atenção e hiperatividade. No entanto, tem efeitos colaterais importantes, tais como dor abdominal, insônia, anorexia, perda de apetite, bem como alguns casos de enfisema precoce grave após abuso da droga. Nosso objetivo foi investigar o desenvolvimento de enfisema pulmonar em ratos submetidos a diferentes doses de metilfenidato.

**TIPO DE ESTUDO E LOCAL:**

Trata-se de estudo experimental realizado em laboratório de uma universidade pública.

**MÉTODOS:**

Dezoito ratos Wistar machos foram divididos em três grupos: Controle (solução salina 0,9%); MP 0.8 (metilfenidato 0,8 mg/kg); MP 1.2 (metilfenidato 1,2 mg/kg). Depois de 90 dias de gavagem diária, os animais sofreram eutanásia e amostras de tecido pulmonar foram preparadas para análise do diâmetro alveolar médio (Lm).

**RESULTADOS:**

Lm foi maior nos grupos 0,8 MP (47,91 ± 3,13, P < 0,01) e MP 1.2 (46,36 ± 4,39, P < 0,05) em comparação com o grupo controle (40,00 ± 3,48).

**CONCLUSÃO:**

O metilfenidato causou aumento no diâmetro alveolar de ratos, o que é compatível com enfisema pulmonar humano.

## INTRODUCTION

Attention deficit hyperactivity disorder (ADHD) is one of the most common psychiatric disorders of childhood and may be accompanied by hyperactive behavior followed by attention problems.^1^ The prevalence of ADHD is estimated to be between 3% and 6.3% among school-age children in different geographical areas,^2^ including Brazil, and its prevalence is greater among males.^3^ The treatment consists of psychological and/or psychiatric intervention together with prescription of stimulant drugs. The association of these two therapies is clinically effective and relatively inexpensive.^4^ Methylphenidate is the most widely used psychostimulant drug for ADHD treatment.^2^^,^^5^^,^^6^ It improves attention, concentration and full cognitive function.^7^ The most common adverse effects are abdominal pain, insomnia, anorexia and loss of appetite.^8^ However, the side effects from longer exposure periods have been insufficiently studied,^9^ and there are no conclusive data. Sherman^10^ found a syndrome of pulmonary vascular sclerosis among abusive (intravenous) users of methylphenidate, and reported the cases of six patients with early severe emphysema. An experimental study on rats showed that there was a high concentration of methylphenidate in lung tissue after intraperitoneal injection.^11^

We hypothesized that chronic use of methylphenidate could be related to early pulmonary emphysema through destruction of the alveolar architecture.

## OBJECTIVE

Our aim was to investigate the relationship between methylphenidate and pulmonary emphysema in a rodent model.

## METHODS

This experimental study was approved by our institutional ethics committee on animal use (protocol number 205/2009), and all procedures complied with international standards for animal experimentation. Eighteen male Wistar rats from the animal house of a university were divided into three groups (n = 6): control (0.9% saline solution, 1 ml/kg), MP 0.8 (methylphenidate, 0.8 mg/kg) and MP 1.2 (methylphenidate, 1.2 mg/kg). Based on data from the literature and from our clinical practice, we chose these doses to represent a therapeutic dose (0.8 mg/kg) and an overdose (1.2 mg/kg).^12^^,^^13^

The animals received either saline or methylphenidate daily by means of gavage for 90 days. After this period, the animals were sacrificed by means of a lethal intraperitoneal injection of thiopental sodium (100 mg/kg). The lungs were excised and fixed using intratracheal instillation of 4% paraformaldehyde (at a constant pressure of 20 cmH_2_O). Thus, the trachea was tied off and the lungs were stored in paraformaldehyde solution. After 24 hours, lung samples (5 µm slices) were obtained and subjected to processing for histological analysis on slides stained with hematoxylin-eosin.

The mean linear intercept (Lm) was microscopically determined by using an eyepiece with Weibel reticule (50 straight lines and 100 points; 200 x magnification), in 15 fields per slide.^14^ The Lm was obtained through the following relationship: Lm = Ltot/Li, where Ltot was the total length of the reticule straight lines and Li was the number of intercepts between alveolar septa and reticule straight lines.

Lm values were expressed as mean ± standard deviation. The Shapiro-Wilk test was used to investigate whether the data presented normal distribution. Comparisons between groups was made using analysis of variance (ANOVA) with the Tukey post-test. A P value less than 0.05 was considered significant.

## RESULTS

**Figure 1.**
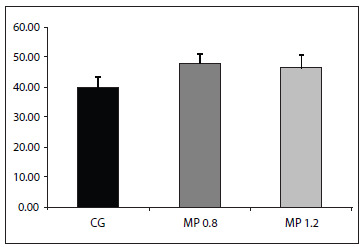
Lm of animals treated either with saline solution (CG, control group) or different doses of methylphenidate (MP 0.8 and MP 1.2).

**Figure 2.**
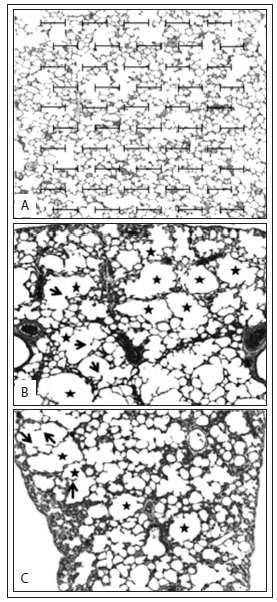
Photomicrographs of lung parenchyma of rats for determination of mean alveolar diameter (Lm) using a Weibel reticule. (A) control group: saline solution; (B) methylphenidate 0.8 mg/kg; and (C) methylphenidate 1.2 mg/kg. Note the areas of alveolar space enlargement (stars) due to disruption of alveolar septa, with formation of “drumsticks” (arrows). Hematoxylin-eosin staining, 200 x magnification.

The Lm was greater in all the methylphenidate-treated animals (MP 0.8 = 47.91 ± 3.13, P < 0.01; MP 1.2 = 46.36 ± 4.39, P < 0.05) than in those of the control group (40.00 ± 3.48) (Figure 1). There was no difference between the MP groups. The Lm analysis is illustrated in Figure 2, which shows the alveolar destruction in methylphenidate-treated animals.

## DISCUSSION

In the present study, we used Lm analysis, the gold-standard method for making histological diagnoses of emphysematous pulmonary disease, and observed that there was notable destruction of pulmonary parenchyma in both of the methylphenidate-treated groups.

Until now, the correlation between methylphenidate and pulmonary emphysema^10^ seems not to have aroused any attention within the scientific community. This might be explained by the lack of methodological information about intake and exposure to the drug among users. One group of authors has shown interest in the systemic effects of methylphenidate.^11^ They tested 37 μmol/kg of methylphenidate on rats and observed that there was higher concentration in lung tissue than in heart, liver and brain tissue. However, no controlled studies have been conducted, thus showing the need to investigate these undesirable side effects.

The mechanism through which emphysematous lesions appear is unclear. In a case presented by Sherman et al.,^10^ a link between respiratory changes and intravenous injection of talc (magnesium trisilicate), which is present in methylphenidate pills, was suggested. Relevant information such as length of exposure and dose has not been elucidated.

Although we did not show that the dose was a determining factor for the changes observed, some studies have tested doses that differed from ours. A double-blind study^12^ in which the objective was to evaluate the side effects of different doses of methylphenidate classified the doses of 0.3 mg/kg and 0.5 mg/kg as low and high dose, respectively. In contrast, a meta-analysis by Spencer et al.^13^ found that the doses used in adult patients were smaller than those used in children (0.5 mg/kg/day versus 1.0 mg/kg/day, respectively).

Because of the lack of consensus in the literature on the ideal dosage of methylphenidate, and based upon the maximum daily dose (60 mg),^15^ we decided to test 0.8 mg/kg/day as a therapeutic dose in rats. Furthermore, we considered that application of 1.2 mg/kg/day would be an overdose. With regard to the length of exposure in the proposed protocol, we chose to analyze the Lm after a long period of drug exposure (90 days), considering that drug treatment approaches to ADHD are long-term and may extend until adulthood.^16^^,^^17^

Despite the significance of these results, it is important to highlight that a single variable was analyzed (Lm). Further research needs to be developed in order to address other important variables such as duration of drug exposure, doses and administration routes. In addition, other analytical methodologies should be used such as elastic fiber analysis, clinical imaging examinations and studies on respiratory mechanics, in order to explain the correlation between methylphenidate and pulmonary emphysema.

## CONCLUSION

In this study, administration of methylphenidate caused destruction of the alveolar septa in the lung parenchyma in Wistar rats, which was histologically compatible with pulmonary emphysema.
